# Clinical Virtual Reality tools to advance the prevention, assessment, and treatment of PTSD

**DOI:** 10.1080/20008198.2017.1414560

**Published:** 2017-01-16

**Authors:** Albert ‘Skip’ Rizzo, Russell Shilling

**Affiliations:** ^a^ Institute for Creative Technologies, University of Southern California, Los Angeles, CA, USA; ^b^ Digital Promise Global, Washington, DC, USA

**Keywords:** Virtual Reality, exposure therapy, PTSD, BRAVEMIND, Veterans, service members, resilience, virtual humans, virtual patients, SimCoach, Palabras clave: realidad virtual, terapia de exposición, TEPT, BRAVEMIND, veteranos, miembros de servicio, resiliencia, humanos virtuales, pacientes virtuales, SimCoach, 虚拟现实, 暴露疗法, PTSD, BRAVEMIND, 老兵服务成员, 韧性, 虚拟人, 虚拟病人, SimCoach, • The article presents the use of Virtual Reality (VR) as a clinical tool to address the assessment, prevention, and treatment of posttraumatic stress disorder (PTSD).• A brief discussion of the definition and rationale for the clinical use of VR is followed by a description of a VR application designed for the delivery of prolonged exposure (PE) for treating Service Members and Veterans with combat- and sexual assault-related PTSD.• Virtual human applications relevant to PTSD are also discussed.• The clinical use of VR is discussed in a larger context, particularly regarding its capability to break down barriers to care.

## Abstract

Numerous reports indicate that the incidence of posttraumatic stress disorder (PTSD) in Operation Enduring Freedom/Operation Iraqi Freedom/Operation New Dawn (OEF/OIF/OND) military personnel has created a significant behavioural healthcare challenge. These findings have served to motivate research on how to better develop and disseminate evidence-based treatments for PTSD. The current article presents the use of Virtual Reality (VR) as a clinical tool to address the assessment, prevention, and treatment of PTSD, based on the VR projects that were evolved at the University of Southern California Institute for Creative Technologies since 2004. A brief discussion of the definition and rationale for the clinical use of VR is followed by a description of a VR application designed for the delivery of prolonged exposure (PE) for treating Service Members (SMs) and Veterans with combat- and sexual assault-related PTSD. The expansion of the virtual treatment simulations of Iraq and Afghanistan for PTSD assessment and prevention is then presented. This is followed by a forward-looking discussion that details early efforts to develop virtual human agent systems that serve the role of virtual patients for training the next generation of clinical providers, as healthcare guides that can be used to support anonymous access to trauma-relevant behavioural healthcare information, and as clinical interviewers capable of automated behaviour analysis of users to infer psychological state. The paper will conclude with a discussion of VR as a tool for breaking down barriers to care in addition to its direct application in assessment and intervention.

## Abstract (es)

Numerosos informes indican que la incidencia del trastorno por estrés postraumático (TEPT) en el personal militar de Operación Libertad Duradera/Operación Libertad Iraquí/Operación Nuevo Amanecer (OEF/OIF/OND, por sus siglas en inglés) ha generado un reto significativo en la atención de la salud conductual. Estos hallazgos han servido para fomentar la investigación sobre cómo desarrollar y difundir mejor los tratamientos basados en la evidencia para el TEPT. El presente artículo presenta el uso de la Realidad Virtual (RV) como herramienta clínica para abordar la evaluación, la prevención y el tratamiento del TEPT, basado en los proyectos de realidad virtual que se han desarrollado en el Instituto de Tecnologías Creativas de la Universidad del Sur de California desde 2004. Una breve discusión de la definición y la justificación para el uso clínico de la RV va seguida de una descripción de una aplicación de RV diseñada para suministrar exposición prolongada (EP) para el tratamiento de miembros de servicio (MS) y veteranos con TEPT relacionado con el combate y la agresión sexual. A continuación, se presenta la expansión de las simulaciones virtuales de tratamiento de Iraq y Afganistán para la evaluación y la prevención del TEPT. Esto es seguido por una discusión prospectiva que detalla los primeros esfuerzos para desarrollar sistemas virtuales de agentes humanos que cumplen la función de pacientes virtuales para formar a la próxima generación de proveedores clínicos, como guías de atención médica que pueden utilizarse para respaldar el acceso anónimo a información de salud conductual relevante en relación al trauma, y como entrevistadores clínicos capaces de realizar un análisis del comportamiento automatizado de los usuarios para inferir el estado psicológico. El documento concluirá con una discusión sobre la realidad virtual como herramienta para eliminar las barreras a la atención médica, además de su aplicación directa en la evaluación y la intervención.

## Abstract (zh)

大量报道指出创伤后应激障碍（PTSD）在‘持久自由据军事行动/伊拉克自由军事行动/新曙光军事行动（OEF/OIF/OND）’的军队人员中的发生率已经给行为医疗护理带来了不容忽视的的挑战。这些发现有助于鼓励对如何更好发展和推广 PTSD 治疗进行研究。这篇文章基于南加里佛利亚大学创造性技术研究所从2004年开始 VR 项目，展示了使用虚拟现实（VR）作为临床工具来解决评估、干预和 PTSD 治疗的问题。首先是关于 VR 临床使用的定义和原理的简短讨论，然后描述VR 在延长暴露治疗（PE）的应用。这个治疗用于患有战争和性侵犯相关的 PTSD 的服务人员（SMs）和老兵。之后呈现了对这项技术扩展应用：使用虚拟治疗模拟伊拉克和阿富汗场景进行 PTSD评估和干预。这之后，有一个前瞻性的讨论详细描述了早期开发虚拟代理系统为虚拟病人服务的努力。这个系统旨在为下一代临床工作者提供训练，并可以用于可匿名获取的创伤相关行为健康护理信息的健康护理指导，同时扮演临床访谈者对使用者进行自动行为分析来推断其心理状态。在结束部分，这篇文章将讨论直接使用VR进行评估和干预以及将它作为打破获取护理帮助障碍的工具。

## 1. Introduction

The physical, emotional, cognitive, and psychological demands of a combat environment place enormous stress on even the best-prepared military personnel. Thus, it is no surprise that the challenging emotional experiences that have been characteristic of the Operation Enduring Freedom/Operation Iraqi Freedom/Operation New Dawn (OEF/OIF/OND) combat theatres have produced significant numbers of Service Members (SMs) and Veterans at risk for developing posttraumatic stress disorder (PTSD) and other psychosocial/behavioural health conditions. For example, as of June 2015, the Defense Medical Surveillance System reported that 138,197 active duty SMs have been diagnosed with PTSD (Fischer, 2015). In a meta-analysis across studies since 2001, 13.2% of OEF/OIF operational infantry units met criteria for PTSD with the PTSD incidence rising dramatically (25–30%) in infantry units with the highest levels of direct combat (Kok, Herrell, Thomas, & Hoge, 2012). These findings make a compelling case for a continued focus on developing and enhancing the availability of diverse evidence-based treatment options to address this military behavioural healthcare challenge. Moreover, the significant investment and effort expended in these efforts, supported by the US Department of Defense, may provide value for informing other VR trauma-focused work in civilian populations where a significant need exists (PTSD United Statistics, 2017). Such populations include victims of terrorist attacks, physical/sexual assault, motor vehicle accidents, and extreme weather events.

One emerging area of research is in the clinical use of Virtual Reality (VR) simulation technology as a tool for delivering evidence-based approaches for the assessment, prevention, and treatment of PTSD. Although in recent times the popular media has lavishly reported on VR’s potential impact on all elements of our evolving digital culture, and has created the impression that VR is a novel technology, the reality is that VR is not a new concept, and many of its developmental roots are traceable to the 1980s and 1990s (Schnipper et al., 2015). Moreover, since the 1990s a significant scientific literature has evolved, almost under the radar, reporting many positive outcomes across a range of clinical applications that have leveraged the assets provided by VR (Botella, Serrano, Baños, & García-Palacios, 2015; Dascal et al., 2017; Freeman et al., 2017; Hoffman et al., 2011; Howard, 2017; Maples-Keller, Yasinski, Manjin, & Rothbaum, 2017; Morina, Ijntema, Meyerbröker, & Emmelkamp, 2015; Rizzo, 1994; Rizzo et al., 2006; Rizzo, Buckwalter, & van der Zaag, 2002, 2015a; Rizzo et al., 2010, in press, 2017, 2015b; Slater & Sanchez-Vives, 2016). Within that context, the present paper will summarize the ways that researchers and clinicians have employed VR to create relevant simulations that can be applied to the prevention, assessment, and treatment of PTSD. Some of the discussion in the current paper includes topics that have been discussed in previous papers, which may be consulted for deeper analysis (Rizzo et al., 2013; Rizzo, Buckwalter, & Neumann, 1997; Rizzo et al., 2010; Rizzo & Koenig, in press; Rizzo et al., 2017; Rizzo, Schultheis, Kerns, & Mateer, 2004).

By its nature, VR applications can be designed to simulate naturalistic environments. Within these virtual environments, researchers and clinicians can present ecologically relevant stimuli embedded in a meaningful and familiar simulated context. VR simulation technology also offers the potential to create systematic human testing, training, and treatment environments that allow for the precise control of complex, immersive, dynamic three-dimensional (3D) stimulus presentations, within which sophisticated interaction, behavioural tracking, user response, and performance recording is possible. When combining these assets within the context of ecologically relevant VR scenarios, a fundamental advancement emerges in how human assessment and intervention can be addressed in many clinical and research disciplines.

VR-based testing, training, and treatment approaches that would be difficult, if not impossible, to deliver using traditional methods have now been developed that take advantage of the assets available with VR technology (Rizzo et al., 2004; Rizzo & Koenig, in press). This unique match between VR technology assets and the needs of various clinical application areas has been recognized by a determined and expanding group of researchers and clinicians. This recognition of the potential impact of VR technology has led to the emergence of a significant, albeit still maturing, research literature that documents the many clinical and research targets where VR can add value relative to traditional assessment and intervention methods. A short list of the areas where Clinical VR has been usefully applied includes fear reduction in persons with specific phobias (Morina et al., 2015; Opris et al., 2012; Parsons & Rizzo, 2008; Powers & Emmelkamp, 2008), treatment for posttraumatic stress disorder (Beidel, Frueh, Neer, & Lejuez, 2017; Botella et al., 2015; Difede & Hoffman, 2002; Difede et al., 2007, 2014; Maples-Keller et al., 2017; McLay et al., 2011; Rizzo et al., 2010, 2013, 2017; Rothbaum, Hodges, Ready, Graap, & Alarcon, 2001; Rothbaum et al., 2014), cue-exposure for addiction and relapse prevention (Hone-Blanchet, Wensing, & Fecteau, 2014; Yoon et al., 2014), depression (Falconer et al., 2016), paranoid delusions (Freeman et al., 2016), discomfort reduction in cancer patients undergoing chemotherapy (Schneider, Kisby, & Flint, 2010), acute pain reduction during wound care and physical therapy with burn patients (Hoffman et al., 2011), other painful procedures (Gold et al., 2006; Mosadeghi, Reid, Martinez, Rosen, & Spiegel, 2016), body image disturbances in patients with eating disorders (Riva, 2011), navigation and spatial training in children and adults with motor impairments (John, Pop, Day, Ritsos, & Headleand, 2017), functional skill training and motor rehabilitation in patients with central nervous system dysfunction (e.g. stroke, traumatic brain injury, spinal cord injury, cerebral palsy, multiple sclerosis, etc.) (Deutsch & McCoy, 2017; Howard, 2017; Klamroth-Marganska et al., 2014; Lange et al., 2012; Merians et al., 2010), and for the assessment and rehabilitation of attention, memory, spatial skills, and other cognitive functions in both clinical and unimpaired populations (Bogdanova, Yee, Ho, & Cicerone, 2016; Matheis et al., 2007; Parsons, Rizzo, Rogers, & York, 2009; Pugnetti et al., 1995; Rizzo, 1994; Rizzo et al., 2006; Valladares-Rodriguez et al., 2016).

To do this, Clinical VR scientists have constructed virtual airplanes, skyscrapers, spiders, battlefields, social settings, beaches, fantasy worlds, and the mundane (but highly relevant) functional environments of the schoolroom, office, home, street, and supermarket. In essence, VR environments mimicking real or imagined worlds can be applied to engage users in simulations that support the aims and mechanics of a specific clinical assessment or therapeutic approach. As a result, there is a growing consensus that VR has now emerged as a promising tool in many domains of research (Bohil, Alicea, & Biocca, 2011) and clinical care (Norcross, Pfund, & Prochaska, 2013). Based on the parallel advances in research and technology, VR has now emerged as a promising tool in many domains of clinical care and research.

## 2. Virtual Reality definitions and technology

The concept and definition of VR has been subject to debate by scientists and clinicians over the years. VR has been very generally defined as a way for humans to visualize, manipulate, and interact with computers and extremely complex data (Aukstakalnis & Blatner, 1992). From this baseline perspective, VR can be seen as an advanced form of human–computer interaction (Rizzo et al., 1997) that allows a user to more naturally interact with computer-generated 3D graphic or photographic simulations of real or imaginary environments beyond what is typically afforded with standard mouse and keyboard interface devices. Moreover, some VR formats enable users to become immersed within synthetic computer-generated virtual environments. However, VR is not defined or limited by any one technological approach or hardware set up. The creation of an engaged VR *user experience* can be accomplished using combinations of a wide variety of interaction devices, sensory display systems, and content presented in the virtual environment. Thus, there are two general variations for how VR can be created and used.

*Non-immersive VR* is the most basic format and is similar to the experience of someone playing a modern computer or console videogame. Content is delivered on a standard flat-screen computer monitor or TV with no occlusion of the outside world. Users interact with 3D computer graphics using a gamepad, a joystick, specialized interface devices, as well as basic mouse or keyboard. Modern computer games that support user interaction and navigation within such 3D worlds, even though presented on a flat-screen display, can technically be referred to as VR environments. Non-immersive VR is also commonly used to support interaction with Virtual Human (VH) agents. These types of applications have focused on VH conversational interactions for training novice clinicians using virtual patients (Talbot, Sagae, John, & Rizzo, 2012; Rizzo et al., 2016a), providing users with a private, online VH context for accessing/discussing health care information (Bickmore, Utami, Matsuyama, & Paasche-Orlow, 2016; Rizzo et al., 2013, 2015b), and for automating a clinical assessment with VH interviewer (Rizzo et al., 2016b).

*Immersive VR* can be produced by the integration of computers, head-mounted displays (HMDs), body-tracking sensors, specialized interface devices, and 3D graphics. These set-ups allow users to operate in a computer-generated simulated world which changes in a natural or intuitive way with head and body motion. Using an HMD that occludes the user’s view of the outside world, an engaged immersive virtual experience employs head- and body-tracking technology that senses the user’s position and movement and sends that information to a computing system that can update the sensory stimuli presented to the user in near real-time, contingent on user activity. This serves to create the illusion of being immersed ‘in’ a virtual space, within which users can interact. When immersed within computer-generated visual imagery and sounds of a simulated virtual scene, user interaction produces an experience that corresponds to what the individual would see and hear if the scene were real. Another less common method for producing immersive VR experiences uses stereoscopic projection screens arrayed around a user in various configurations (Baños et al., 2009).

Regardless of the technical approach, the key aim of these immersive systems is to perceptually replace the outside world with the virtual world to psychologically engage users with simulated digital content designed to create a specific user experience. Immersive VR (most commonly delivered in a HMD) is typically the choice for applications where a controlled stimulus environment is desirable for constraining a user’s perceptual experience within a specific synthetic world. This format has been often used in Clinical VR applications for delivering exposure therapy for anxiety disorders and PTSD, analgesic distraction for patients undergoing acutely painful medical procedures, and in the cognitive assessment of users to measure performance under a range of systematically delivered challenges and distractions.

## 3. Virtual Reality prolonged exposure for PTSD

Among the many approaches that have been used to treat persons with PTSD, prolonged exposure (PE) therapy has significant scientific support for its therapeutic efficacy (Bryant, 2005; IOM, 2008; 2012; Maples-Keller et al., 2017; Rothbaum, 2001). PE is a form of individual psychotherapy based on the Foa and Kozak (1986) emotional processing theory, which posits that phobic disorders and PTSD involve pathological fear structures that are activated when information represented in the structures is encountered. Successful treatment requires emotional processing of the fear structures in order to modify their pathological elements so that the stimuli no longer invoke fear, and any method capable of activating the fear structure and modifying it in a safe environment would be predicted to improve symptoms of anxiety. The proposed mechanisms for symptom reduction involve activation and emotional processing, extinction/habituation of the anxiety, cognitive reprocessing of pathogenic meanings, the learning of new responses to previously feared stimuli, and ultimately an integration of corrective non-pathological information into the fear structure (Foa & Hearst-Ikeda, 1996). In practice, such treatment typically involves the graded and repeated imaginal reliving and narrative recounting of the traumatic event within the therapeutic setting. This approach is believed to provide a low-threat context where the client can begin to confront and therapeutically process the emotions that are relevant to a traumatic event as well as de-condition the learning cycle of the disorder via an extinction learning process. While the efficacy of imaginal PE has been established in multiple studies with diverse trauma populations (Bryant, 2005; Rothbaum & Schwartz, 2002; Van Etten & Taylor, 1998), many patients are unwilling or unable to effectively visualize the traumatic event, and this may result in treatment failure (Difede & Hoffman, 2002). In fact, avoidance of reminders of the trauma is inherent in PTSD and is one of the cardinal symptoms of the disorder.

To address this problem, researchers have explored the use of VR as a tool to deliver exposure therapy (VRET). The rationale for this is straightforward. The VR delivery of an evidence-based PE protocol is seen as a way to immerse users in simulations of trauma-relevant environments in which the emotional intensity of the scenes can be precisely controlled by the clinician to customize the pace and relevance of the exposure for the individual patient. In this fashion, VRET offers a way to circumvent the natural avoidance tendency by directly delivering multi-sensory and context-relevant cues that aid in the retrieval, confrontation, and processing of traumatic experiences. Within a VR environment, the hidden world of the patient’s imagination is not exclusively relied upon, in effect taking some of the weight off their shoulders. VR also provides an objective and consistent format for documenting the sensory stimuli that the patient is exposed to that is not possible when operating within the unseen world of the patient’s imagination. Previous success in similarly using VRET for persons with other anxiety disorders, such as specific phobias, has been documented in multiple independent meta-analyses and reviews of the literature (Morina et al., 2015; Opriş et al., 2012; Parsons & Rizzo, 2008; Powers and Emmelcamp, 2008). Multiple studies report positive outcomes using VRET with non-OEF/OIF PTSD clients (i.e. World Trade Center survivors and Vietnam Veterans) who were unresponsive to a previous course of *imaginal-only* PE treatment (Difede et al., 2007; Difede & Hoffman, 2002; Rothbaum et al., 2001).

The first effort to apply VRET for PTSD with military populations began in 1997 when researchers at Georgia Tech and Emory University began testing the *Virtual Vietnam* VR scenario with Vietnam veterans diagnosed with PTSD (Rothbaum et al., 1999, 2001). This occurred over 20 years after the end of the Vietnam War. During those intervening years, in spite of valiant efforts to develop and apply traditional psychotherapeutic and pharmacological treatment approaches to PTSD, the progression of the disorder in some veterans significantly impacted their psychological well-being, functional abilities, and quality of life, as well as that of their families and friends. This initial effort yielded encouraging results in a case study of a 50-year-old male Vietnam veteran meeting DSM IV-R criteria for PTSD (Rothbaum et al., 1999). Results indicated posttreatment improvement on all measures of PTSD and maintenance of these gains at a six-month follow-up, with a 34% decrease in clinician-rated symptoms of PTSD and a 45% decrease in self-reported symptoms of PTSD. This case study was followed by an open clinical trial with Vietnam veterans (Rothbaum et al., 2001). In this study, 16 male veterans with PTSD were exposed to two HMD-delivered virtual environments, a virtual clearing surrounded by jungle scenery and a virtual Huey helicopter, in which the therapist controlled various visual and auditory effects (e.g. rockets, explosions, day/night, shouting). After an average of 13 exposure therapy sessions over 5–7 weeks, there was a significant reduction in PTSD and related symptoms. For a more current and detailed review of the use of VR-enhanced extinction approaches to the treatment of phobias and PTSD, the reader is directed to Maples-Keller et al. (2017). (For more information, see the nine-minute Virtual Vietnam Documentary video at: http://www.youtube.com/watch?v=C_2ZkvAMih8).

## 4. Virtual Iraq/Afghanistan and the BRAVEMIND VRET systems

In anticipation of impending military behavioural health needs, and supported by a clear theoretical rationale and the extant literature (Maples-Keller et al., 2017), the USC Institute for Creative Technologies developed an initial prototype Virtual Iraq VRET system in 2004 for conducting user tests to determine feasibility (https://www.youtube.com/watch?v=zTtaK6mK3_c). This was followed by the creation of a four-scenario clinical system ‘Virtual Iraq/Afghanistan’ developed during 2005–2007 and funded by the US Office of Naval Research (https://www.youtube.com/playlist?list=PLMuMO5eoYy_C4BOBK8eW72wdtQqJvhTxa). The system was the product of both theory-driven design and iterative user-centred feedback cycles with OEF/OIF service members to maximize its credibility/relevance/usability for clinical users. Pre-clinical user-testing was conducted at Fort Lewis, Washington, and within an Army Combat Stress Control Team in Iraq (Reger, Gahm, Rizzo, Swanson, & Duma, 2009). This feedback from non-diagnosed SMs (and later by initial clinical users) provided essential input for an iterative user-centred design process that continues to evolve the clinical VRET system to the current day.

In 2011, based on the early promising outcomes of the initial Virtual Iraq/Afghanistan application, the US Army funded the development of an updated and expanded version of the system. Rebuilt from the ground up using the state-of-the-art *Unity Game Engine*, the system is now referred to as *BRAVEMIND* (https://www.youtube.com/playlist?list=PLMuMO5eoYy_CnHj35tDGPYfR3fLDY0AyG). One of the primary goals for this effort was to increase the diversity of the VR scenario content and improve the customizability of scenario and stimulus delivery to better address the needs of clinical users who presented with a diverse range of trauma experiences. The four original 2007 environments were completely rebuilt and 10 additional scenarios were added for a total of 14, including: separate Iraq and Afghanistan cities, a rural Afghan village, an industrial zone, a roadway checkpoint, slum and high-end residential areas, a mountainous forward operating base, and a hospital receiving area modelled after one at Bagram Airfield. The general design of the BRAVEMIND treatment environment consists of a series of selectable virtual scenarios designed to represent relevant contexts for VR exposure therapy with OIF/OEF SMs and Veterans. In addition to the visual stimuli presented in the VR head mounted display, directional 3D audio, vibrotactile, and olfactory stimuli of relevance can be delivered. Clients’ experiences of VR scenarios and stimuli are controlled by a clinician in real time via a separate ‘Wizard of Oz’ (WoZ) control panel, while in full audio contact with the client during exposure. The WoZ control panel is essentially a clinician interface computer screen that allows the clinician to control the stimulus content presented in real time to a user within the VR simulations. It consists of buttons on a computer screen that can be enabled/disabled with a mouse click, to pace the customized delivery of relevant trigger stimuli to the user. This video shows a clinician operating the WoZ system in a mock demonstration at the 45 second mark (https://www.youtube.com/watch?v=nrgUPVFY44o&list=PLMuMO5eoYy_CnHj35tDGPYfR3fLDY0AyG). This level of stimulus delivery/control is required to support a clinician’s ability to foster the anxiety modulation needed for therapeutic exposure to produce extinction learning and emotional processing in a fashion customized to the patient’s past experience and ongoing treatment progress. The BRAVEMIND update was informed by drawing on the vast amount of user feedback generated from both client and clinician feedback from use of the previous 2007 Virtual Iraq/Afghanistan system. A detailed description of the Virtual Iraq/Afghanistan system and the methodology for a standard VRET clinical protocol can be found elsewhere (Rothbaum, Difede, & Rizzo, 2008); technical/clinical details on the current BRAVEMIND system can be found in Rizzo et al. (2017).

## 5. VRET research outcomes

Early clinical tests of the Virtual Iraq/Afghanistan system produced promising results. Initially, three published case studies reported positive results using this system (Gerardi, Rothbaum, Ressler, Heekin, & Rizzo, 2008; Reger & Gahm, 2008; Rizzo et al., 2007). In the first open clinical trial (Rizzo et al., 2010), analyses of 20 active duty treatment completers (19 male, one female, Mean Age = 28, Age Range: 21–51) produced positive clinical outcomes. For this uncontrolled feasibility trial, mean pre/post PCL-M (Blanchard, Jones-Alexander, Buckley, & Forneris, 1996) scores decreased in a statistical and clinically meaningful fashion: 54.4 (*SD* = 9.7) to 35.6 (*SD* = 17.4). Paired pre/post *t*-test analysis showed these differences to be significant (*t* = 5.99, *df* = 19, *p* < .001). Correcting for the PCL-M no-symptom baseline of 17 indicated a greater than 50% decrease in symptoms and 16 of the 20 completers no longer met PCL-M criteria for PTSD at post treatment. Mean Beck Anxiety Inventory (Beck, Epstein, Brown, & Steer, 1988) scores significantly decreased 33% from 18.6 (*SD* = 9.5) to 11.9 (*SD* = 13.6), (*t* = 3.37, *df* = 19, *p* < .003) and mean PHQ-9 (Kroneke and Spitzer, 2002) depression scores decreased 49% from 13.3 (*SD* = 5.4) to 7.1 (*SD* = 6.7), (*t* = 3.68, *df* = 19, *p* < .002). Positive results from uncontrolled open trials are difficult to generalize from and one must be cautious not to make excessive claims based on these early results. However, using an accepted military-relevant diagnostic screening measure (PCL-M), 80% of the treatment completers in the initial VRET sample showed both statistically and clinically meaningful reductions in PTSD, anxiety and depression symptoms, and anecdotal evidence from patient reports suggested that they saw improvements in their everyday life. These improvements were also maintained at three-month post-treatment follow-up. Detailed methodology and results from this trial can be found in Rizzo et al. (2010). In another open clinical trial (Reger et al., 2011) with active duty Army SMs (*n* = 24), the results indicated significant pre/post reductions in PCL-M scores and a large treatment effect size (Cohen’s *d* = 1.17). After an average of seven sessions, 45% of those treated no longer screened positive for PTSD and 62% had reliably improved.

A series of randomized controlled trials (RCTs) were then conducted. In an early trial (Roy, Costanzo, Blair, & Rizzo, 2014), active duty SMs with PTSD (*N* = 19) were randomized to VRET (*n* = 9) or imaginal exposure (*n* = 10). At post-treatment VRET reduced CAPS (Blake et al., 1995) scores (*p *< .05), whereas the imaginal PE showed no significant changes while both groups showed significant change (*p *< .05) on the PCL-M. In another small preliminary quasi-randomized controlled trial (McLay et al., 2011), using a comparable VRET simulation of Iraq as the ICT version described above, seven of 10 participants with PTSD showed a 30% or greater improvement with VR, while only one of nine participants in a ‘treatment as usual’ group showed similar improvement. While the results of these two RCTs are variously limited by small sample sizes, lack of blinding, use of a single therapist, and, in the case of McLay et al. (2011), the VRET comparison was with a set of relatively uncontrolled usual care conditions, these findings added to the incremental evidence in support of the use of VRET for combat-related PTSD. More equivocal findings were reported in Reger et al. (2016) in a RCT comparing VRET, PE, and a waitlist control with active duty OIF/OEF soldiers with PTSD (*n* = 162). Although both VRET and PE demonstrated significantly more improvement on PTSD and depressive symptoms relative to waitlist control, no significant differences were observed between VRET and PE at post-treatment. Moreover, greater improvement in PTSD symptoms at the three- and six-month follow-up was found with PE. One possible explanation for these follow-up results, that are in sharp contrast to previous findings indicating strong durability of VRET treatment gains (Difede et al., 2014; Rizzo et al., 2010; Rothbaum et al., 2014), is that the study employed the more limited four scenario Virtual Iraq/Afghanistan system that may have provided less relevant content to this specific group of active duty SM clients. Previous feedback from clinicians using this system indicated that, when the client’s trauma experience was not well matched to the available content in this initial system, clinicians would shift to imaginal PE. Such feedback informed the design of the BRAVEMIND system with its expansion to 14 diverse scenarios and clinical trials with that version of the system are underway.

There are also reports from two other large scale VRET trials, which have examined the augmentation of the traditional exposure component with additional psychosocial treatment (Beidel et al., 2017) and with a pharmacological supplement (Rothbaum et al., 2014). Beidel et al. (2017) combined VRET with Trauma Management Therapy (TMT) within an intensive daily outpatient programme conducted over three weeks. VRET was delivered each morning and TMT (Turner, Beidel, & Frueh, 2005) was conducted each afternoon as a highly-structured group intervention that focused on social reintegration, anger management/problem solving training, and brief behavioural activation for depression. With an analysed sample size of 102 and a 2% dropout rate, the authors reported a 2.06 effect size, with 65.9% no longer meeting diagnostic criteria for PTSD. Similar positive effects were reported in other clinical domains and treatment gains were maintained at six-month follow-up. Although it is impossible to determine the differential effects of VRET vs. the psychosocial TMT components, the results from the combination of these approaches in an intensive format are promising, especially in light of recent criticism of prolonged exposure approaches (Steenkamp, 2016).

Finally, Rothbaum et al. (2014) compared the effects of five VRET sessions augmented by D-cycloserine (DCS), which has been found to facilitate extinction in other fear-based disorders (Ressler et al., 2004), alprazolam, and placebo in a study with 156 OIF/OIF Veterans with PTSD. Although there were no differences in treatment outcome across medication conditions, with the exception of posttreatment and three-month follow-up CAPS scores indicating that the alprazolam group showed a higher rate of PTSD than the placebo group, PTSD symptoms significantly improved across all conditions at posttreatment and at the three-, six-, and 12-month follow-ups. Moreover, VRET resulted in improvement in psychobiological measures of startle and cortisol reactivity to a trauma relevant scene (Norrholm et al., 2016), providing further support for the effectiveness of this form of extinction training using VR (Maples-Keller et al., 2017).

An ongoing RCT using the BRAVEMIND system is nearing completion at the time of this writing investigating the additive value of DCS with VRET and PE (Difede, Rothbaum, & Rizzo, 2010). Recent evidence of both VRET and DCS effectiveness was been reported by Difede et al. (2014) in a clinical trial with World Trade Center PTSD clients. In a double-blinded controlled comparison between VRET+DCS and VRET+Placebo, both groups had clinically meaningful and statistically significant positive outcomes with the DCS group achieving equivalent gains with fewer sessions. This finding is in contrast with two reports that found no additive value when adding DCS to imaginal PE for PTSD treatment in civilian (de Kleine, Hendriks, Kusters, Broekman, & van Minnen, 2012) and military (Litz et al., 2012) groups. The current ongoing RCT (Difede et al., 2010) will be important for determining whether DCS will differentially improve PTSD treatment outcomes across PE and VRET conditions in view of previously reported mixed findings in this literature.

While not detailed in this paper, interested readers are invited to examine reports from other groups that have conducted research with VR content created by other developers or who have implemented alternative (non-PE-based) treatment approaches. One study reported a significant decrease over time on the CAPS Criterion C (avoidance/numbing symptoms) in the VR group (*F*(1,20) = 6.03, *p* = .02) implementing a PE approach using a VR system that was limited to one Humvee driving scenario (Miyahira et al., 2012). Wood et al. (2008) has reported positive findings using another VR system implementing a systematic desensitization approach driven by clinician administered pacing of the trigger stimulus presentation based on real time physiological monitoring of the user (Wood et al., 2008). Along similar lines, Ćosić, Popović, Kukolja, Horvat, and Dropuljić (2010) presents an elegant methodology for using advanced computing to generate automatic changes in stimulus presentations based on real time analysis of physiological changes in the user.

In conclusion, the overall trend of the published findings is encouraging for the view that VRET can be safely applied clinically and may be an effective approach for delivering an evidence-based treatment (PE) for PTSD. At the least, with the exception of the follow-up data from the Reger et al. (2016) trial, these studies suggest that VRET is as efficacious as traditional PE, although more research is needed in the form of high quality RCTs using the current BRAVEMIND system before this can be fully ascertained.

## 6. VRET expansion for combat medics/corpsman and military sexual trauma

The 2011 rebuild of the BRAVEMIND system provided an updated software architecture that supports the flexible and efficient expansion of the system’s content and functionality. This has supported the creation of new customizable content for conducting VRET with a wider range of relevant trauma experiences. The BRAVEMIND VRET system is now being further evolved to address the unique therapeutic needs of combat medics/corpsmen and of persons who have experienced military sexual trauma (MST) with PTSD.

VRET for Combat Medics/Corpsman was hypothesized to require specialized VR content that is more relevant to the unique challenges that these groups face. Thus, with funding from the *Oakley Infinite Hero Foundation*, the existing BRAVEMIND scenarios were extended to include more wounded virtual humans that can display a range of wounds/burns and manifest realistic injury behaviours. Helicopter insertion and extraction scenarios and a Bagram Air Force Base hospital setting for medic/corpsmen ‘first receivers’ were developed. This effort required the creation of significant new graphic art, motion capture animation, and airborne vehicle integration in order to offer relevant VRET for combat medics/corpsmen with PTSD. These elements are included as options in the currently available system, but no outcome data has been reported thus far on its specific use.

PTSD can result from exposure to actual or threatened death, serious injury, or sexual violation. This is of particular relevance for SMs who may face trauma from both the threat that is naturally inherent in the combat theatre, as well as from the possible additive occurrence of sexual violations from within the ranks. Thus, MST that is experienced as a threat or result of an occurrence of a sexual violation/assault within a military context can produce additional risk for the development of PTSD in a population that is already at high risk due to the existing occupational hazards present in the combat environment. This is an issue of significant concern for the Department of Defense (DoD). Thus, in addition to efforts aimed at reducing the incidence of MST with novel education and prevention programmes, the US Army funded the expansion of the BRAVEMIND system to address PTSD due to MST. This involved a significant effort to create new content within the existing BRAVEMIND scenarios such as barracks, tents, other living and work quarters, latrines, and other contexts that have been reported by MST victims as in-theatre locations where their sexual assault occurred. Additionally, stateside military base and civilian contexts were created including barracks, offices, a town bar area, abandoned lots, motel rooms, and civilian automobile settings (https://www.youtube.com/watch?v=ue494drmU0s&t=5s). The system does *not* attempt to recreate a sexual assault, but, rather, sets up the contexts surrounding the assault in which users can be supported in the therapeutic confrontation and processing of MST memories in accordance with the protocol that has been used previously that implements PE within the simulations (Rothbaum et al., 2008). The BRAVEMIND MST is currently being tested in a pilot trial at Emory University. This has not been attempted previously with immersive VRET, although a non-immersive VR system in Europe produced initial positive findings with civilian patients having PTSD due to physical assaults and domestic violence (Baños et al., 2009, 2011). While both men and women can experience MST, the urgent need for this work is underscored by the growing role of women transitioning into full combat roles in the combat theatre, an area that up to now has been primarily the domain of men.

## 7. Beyond VRET: VR for the assessment and prevention of PTSD

### 7.1. Virtual Reality stimuli for PTSD assessment

While VR has been primarily used as a therapeutic tool to enhance the delivery of PE for PTSD, some researchers have begun to explore the re-use of the BRAVEMIND simulation content as stimuli for creating more objective PTS assessment measures. One of the primary challenges for arriving at an accurate diagnosis of PTSD is that the assessment information is typically limited by reliance upon the patient’s subjective reports of his/her traumatic experiences derived from self-report symptom checklists or from structured clinical interview reporting. Many factors can influence the accuracy of this assessment data. Some individuals may under report symptoms because of the stigma of having a mental health disorder, and others may over report symptoms to obtain medical benefits (Gates et al., 2012). Previous research suggests that individuals with PTSD may show differential physiological reactivity in response to specific, emotionally evocative cues; Webb, Vincent, Jin, and Pollack (2015) provide a concise detailing of this literature. Thus, some researchers have attempted to enhance the objective assessment of PTS by combining VR’s capacity to present users with highly controlled, ecologically relevant, and realistic stimulus environments while concurrently recording psychophysiological/biological responses. The use of VR stimuli for this purpose is at an early stage of maturity, but encouraging results have been reported in four studies that directly address the VR/PTSD assessment question (Costanzo et al., 2014; Highland et al., 2015; Norrholm et al., 2016; Webb et al., 2015). In a somewhat related effort, another paper has examined the use of fMRI to assess changes in brain activation following a course of VRET and PE (Roy et al., 2014). This falls in line with a view held by some neuroscientists (Bohil et al., 2011; Tarr & Warren, 2002) that highly controllable VR-generated content may add value as stimuli in brain imaging studies. For a detailed summary of the VR assessment research, see Rizzo et al. (2017).

### 7.2. Virtual Reality resilience training

The current urgency in efforts to address the psychological wounds of war in SMs and Veterans has also driven an emerging focus within the military on emphasizing a proactive approach for better preparing service members for the emotional challenges they may face during a combat deployment to reduce the potential for later adverse psychological reactions such as PTSD and depression. This focus on resilience training prior to deployment represents no less than a quantum shift in military culture and can now be seen emanating from the highest levels of command in the military. For example, in an American Psychologist article, Army General George Casey (2011) makes the case that ‘soldiers can “be” better before deploying to combat so they will not have to “get” better after they return’ (p. 1), and he then calls for a shift in the military ‘to a culture in which psychological fitness is recognized as every bit as important as physical fitness’ (p. 2). This level of endorsement can be seen in practice by way of the significant funding and resources applied to a variety of resilience training programmes across all branches of the US Military (Cornum, Matthews, & Seligman, 2011; Hovar, 2010; Luthar, Cicchetti, & Becker, 2000).

To address the resilience challenge, BRAVEMIND virtual content was retooled to produce a system designed to assess and teach resilience skills, referred to as the *STress Resilience In Virtual Environments* (STRIVE). The STRIVE project aimed to teach resilience via the creation of a set of combat simulations that can be used as virtual contexts for SMs to experientially learn stress reduction tactics and cognitive-behavioural emotional coping strategies *prior* to deployment. This approach involves immersing and engaging SMs within a variety of virtual ‘mission’ episodes where they are confronted with emotionally challenging situations that are inherent to the OEF/OIF/OND combat environment. Interaction by SMs within such emotionally challenging scenarios aims to provide a more meaningful context in which to engage with psychoeducational information and to learn and practice stress reduction tactics and cognitive coping strategies that are believed to better prepare a SM for the psychological challenges that may occur during a combat deployment (see STRIVE episodes: https://www.youtube.com/playlist?list=PLMuMO5eoYy_AgK3ZWznPlTvFNjwKkXK9X).

STRIVE was initially designed as a multi-episode interactive narrative in VR, akin to being immersed within a ‘Band of Brothers’ type storyline of events that could occur during a combat deployment. Within the episodes, SMs get to know the distinct personalities of the virtual human characters in their squad and interact within an immersive digital narrative that employs cinematic strategies for enhancing engagement with the evolving storyline (e.g. strategic use of narration, montage shots, dynamic camera direction). At the midpoint of each of the 15-minute episodes, an emotionally challenging event occurs, designed in part from feedback provided by SMs undergoing PTSD treatment (e.g. seeing/handling human remains, death/injury of a squad member, death/injury of a civilian child, disturbing culturally relative and morally challenging situations, etc.). At that point in the episode, the virtual world ‘freezes in place’ and an intelligent VH ‘mentor’ emerges from the midst of the chaotic VR scenario to guide the user through a variety of resilience-related psychoeducational and self-management tactics, as well as providing rational restructuring exercises for appraising and processing the virtual experience. The VH mentor presents resilience training content that is relevant to the VR context and narrative just experienced and draws on the types of strategies and content that has been endorsed as part of standard classroom-delivered DoD resilience training programmes, as well as content that has been successfully applied in non-military contexts (e.g. humanitarian aid workers, sports psychology, etc.).

In this fashion, STRIVE provides a digital ‘emotional obstacle course’ that can be used as a tool for providing experiences that leverage narrative-based, context-relevant experiential learning of emotional coping strategies under very tightly controlled and scripted simulated conditions. Training in this format is hypothesized to improve generalization to real world situations via a state dependent learning component (Godden & Baddeley, 1980) and further supports resilience by leveraging the learning theory process of latent inhibition. Latent inhibition refers to the delayed learning that occurs as a result of pre-exposure to a stimulus without a consequence (Feldner, Monson, & Friedman, 2007; Lubow & Moore, 1959). Thus, the exposure to a simulated combat context is believed to decrease the likelihood of fear conditioning during the real event (Sones, Thorp, & Raskind, 2011). Moreover, the six episodes created thus far could be used as standardized stimuli which could be presented to users while monitoring psychophysiological and blood biomarker reactivity and recovery levels in an attempt to develop more objective measures of resilience.

In 2014, STRIVE received the *US Army Modeling and Simulation Award for Team Training* as a component of the more comprehensive Squad Overmatch project conducted at Fort. Benning in 2014–2015. In those tests, SMs (*n* = 92) reported high positive ratings (88–100%) of the STRIVE episodes for preparing for and promoting knowledge, visualization, and rehearsal of stressful events to improve survival in a combat environment (Squad Overmatch, 2015). An initial pilot test of the physiologically activating effects of the first four STRIVE mission challenges was conducted with a sample of USC ROTC Cadets (*n* = 39) ( Wellman et al., submitted). Participants who experienced the four STRIVE mission challenges had Fast Fourier transformation of heart rate variability (HRV) measured to infer emotional impact during participation. The change in HRV variability from baseline to the occurrence of each of the pivotal STRIVE end point events revealed significant effects. Significant Low Frequency/High Frequency (LF/HF) ratio changes were seen across all but one event. From these pilot STRIVE studies, we found initial support for positive SM user acceptance/credibility of this format for resilience training content and for physiological activation in response to the pivotal events in each scenario. This bodes well for investigating the feasibility of this approach for teaching resilience strategies. Future technical development aims to leverage a low-cost mobile-phone enabled consumer HMD (*Samsung Gear VR*) to support the widespread dissemination and independent SM practice of this and other military relevant content. This project is noteworthy in that it represents a direct application development effort (resilience training) while also serving as an ‘ultimate Skinner-Box’ for the scientific study of stress reactions using objective physiological assessment measures.

## 8. Virtual humans for clinician training, healthcare information access, and clinical interviewing

There have been dramatic advances in the underlying enabling technologies required for creating believable ‘structural’ VR environments (e.g. combat scenes, homes, classrooms, offices, markets) for clinical applications. The next stage in the evolution of clinical VR involves populating these environments with VH representations that can engage human users in credible and useful interactions. This capability has been around since the late 1990s, but the limitations in voice recognition, natural language processing, artificial intelligence, graphic rendering, and face and gesture animation made the creation of conversational VHs for interaction a costly and challenging process. Until recently, VHs existed primarily in the domain of high-end special effect studios/labs that catered to the film or game industry, far from the reach of those who thought to employ them in clinical health applications. Early efforts to create VH representations appeared in clinical VR scenarios primarily to serve as stimulus ‘props’ to enhance the realism and provocativeness of a virtual world simply by their static presence. For example, VRET applications for the treatment of fear of public speaking and social phobias were successfully deployed using immersive simulations inhabited by ‘still-life’ rendered graphic characters (Anderson, Zimand, Hodges, & Rothbaum, 2005; Klinger et al., 2005; North, North, & Coble, 1998). By adjusting the number and location of such VHs, the intensity of these anxiety-provoking VR contexts could be systematically modulated with the aim to promote fear extinction to improve functioning in the real world with real people. In spite of the primitive nature of these VHs, clients with specific phobias appeared to be especially primed to emotionally react to such representations and, thus, the VHs provided the necessary stimulus elements to be effective in these types of exposure-based cognitive behavioural treatment scenarios.

As the technology evolved, other clinical applications (beyond phobias) began using more animated VHs as stimulus entities to support and train social and safety skills in persons with high functioning autism (Bresnahan et al., 2016; Padget et al., 2006; Rutten et al., 2003) and as distracter stimuli for assessing attention in a virtual classroom (Rizzo et al., 2006). VHs were also used effectively for the conduct of social psychology experiments, essentially replicating and extending findings from studies conducted with real humans on social influence, conformity, racial bias, and social proxemics (Bailenson & Beall, 2006; Blascovich et al., 2002; McCall, Blascovich, Young, & Persky, 2009). The capacity to control the number and provocative nature of animated VHs is a standard feature in the BRAVEMIND PSTD VRET system (Rizzo et al., 2017).

This brings us to the current period, where VH agents can now be created that control computer generated bodies and can interact with users through natural language speech and gesture in virtual environments (Rizzo & Talbot, 2016a; Talbot et al., 2012). Advances in artificial intelligence (AI) can now support the creation of VHs that can engage users in rich conversations (Morbini et al., 2014), recognize nonverbal cues (Rizzo et al., 2016b, 2015b; Scherer et al., 2014), improve interactional rapport (Park, Scherer, Gratch, Carnevale, & Morency, 2013), reason about social and emotional factors (Gratch & Marsella, 2004), and synthesize human communication and nonverbal expressions (Morency, de Kok, & Gratch, 2008). No longer at the level of a prop to add context or minimal faux interaction in a virtual world, VH agents can be designed to perceive and act in a 3D virtual world, engage in face-to-face spoken dialogues with real users, and, in some cases, they are capable of exhibiting human-like emotional reactions.

These advances in VH systems could have significant impact on the future of clinical training and provide unique methods for addressing the needs of persons suffering from the experience of trauma. For example, in 2007, our centre at the University of Southern California (USC) leveraged military-funded VH technology originally designed for leadership roleplay training, to create virtual patients for training novice clinicians the skills required for clinical interviewing with difficult clients (Kenny, Rizzo, Parsons, Gratch, & Swartout, 2007; Parsons et al., 2008; Rizzo et al., 2016a; Talbot et al., 2012). The first USC virtual patient project was designed to provide medical students with practice interviewing with a VH sexual trauma client (https://www.youtube.com/watch?v=jy1NKDz47aQ&t=14s) and the system was later evolved to train social workers how to assess suicide risk in SMs and Veterans (https://www.youtube.com/watch?v=CQTEcJJ_RhY). This work has now led to the development of an authoring system that clinical educators can access online to create training cases relevant to any clinical condition drawing from a library of 40 VHs that represent varied age, gender, and ethnic backgrounds (https://www.youtube.com/watch?v=PdgwI_l9fpM&t=4s).

VHs can also serve in the role of an online and always available mental healthcare support agent. For example, the SimCoach project (https://www.youtube.com/watch?v=PGYUqTvE6Jo&t=7s) (Rizzo et al., 2013, 2015b) resulted in a web-accessible online conversational guide for promoting access to psychological healthcare information (www.simcoach.org). The system was designed for use by SMs and Veterans and arose from the recognition that certain ‘barriers to care’ lessened the likelihood that this population would seek psychological care from a live provider. Whether due to stigma and/or a fear that such help-seeking could have a negative impact on their future career options within the military, the SimCoach support agent was designed to provide a private and anonymous space for users to interact with a conversational VH to access information about PTSD. SimCoach characters are able to solicit basic anonymous background information about the user’s history and conduct short screening questionnaires that assess a user’s clinical/psychosocial concerns. Armed with this information, the SimCoach can provide low-level advice/support, direct the user to relevant online content, and facilitate the process of seeking appropriate care with a live clinical provider, if that option is chosen by the user. While much of the information provided by SimCoach is similar to what could be obtained from text-based websites such as *WebMD* or *AfterDeployment*, the use of conversational interaction with a highly approachable virtual character may serve to create rapport, establish trust, and encourage some users to seek the help they need within this private and anonymous interaction. The SimCoach virtual support agent does not deliver diagnosis or treatment, nor do they aim to replace human providers and experts. Rather, SimCoach characters provide users with an accessible and anonymous way engage in a dialogue about mental healthcare concerns as a safe approach for people who may initially be hesitant to seek care with a live provider. Initial user feedback has been positive (Rizzo et al., 2013) and the system has been iteratively evolved with new information/functionality based on user interaction with the system. As well, the system is highly authorable and has been repurposed for other clinical activities (Rizzo et al., 2015b).

An expansion of the SimCoach system referred to as ‘SimSensei’ is a VH interviewing platform that integrates off-the-shelf sensors (i.e. webcams, Microsoft Kinect, microphone) to capture and interpret real-time audiovisual behavioural signals from users interacting with the VH system. The system was specifically designed for clinical interviewing and health care support by providing a face-to-face interaction between a user and a VH that can automatically react to the inferred state of the user through analysis of behavioural signals gleaned from the user’s facial expressions, body gestures, and vocal parameters. Akin to how non-verbal behavioural signals have an impact on human-to-human interaction and communication, SimSensei aims to capture and infer user state from signals generated from user non-verbal communication. The SimSensei uses this information to adjust follow-up responses aimed at improving engagement with the user and to quantify user state from the data captured across a 20-minute interview (https://www.youtube.com/watch?v=Yw1c5h_p6Dc&t=8s and https://www.youtube.com/watch?v=_uYokWUSark&t=34s). Research with SimSensei thus far suggests that it may be useful for conducting clinically-oriented interviews within a safe non-judgmental context that encourages more honest disclosure in interviewees. In one controlled study with civilians, users reported less concern about being evaluated and verbally disclosed/behaviourally displayed more sadness in an interview with a SimSensei VH agent compared to users interacting with a VH avatar that they believed was being operated by a human-in-the-loop ‘Wizard of Oz’ controller (Lucas et al., 2014). More recently, results from of sample of military SMs who were interviewed by the SimSensei clinical interviewer before and after a deployment to Afghanistan indicated that SMs revealed more PTSD symptoms to the VH than they reported on both the standard and anonymized Post Deployment Health Assessment upon return home (Rizzo et al., 2016b). Pre/post deployment facial expression analysis indicated more sad expressions and fewer happy expressions at post deployment.

Thus, VHs now are capable of fostering interactions with real people that can address a wide variety of clinical concerns. There is a growing literature in this area and it is not hard to see the power of VH applications to foster roleplay training that targets social interaction, anger management, relapse prevention for addiction, and many other areas where clinical populations could benefit from low social risk interaction with a non-judgmental VH (Albright, Adam, Serri, Bleeker, & Goldman, 2016; Bickmore et al., 2016; Rizzo et al., 2016b; Tegos, Demetriadis, Papadopoulos, & Weinberger, 2016; Zhang, Bickmore, & Paasche-Orlow, 2017). Although some authors have expressed legitimate concerns about the role of VH ‘automation’ supplanting the role of clinicians (Innes & Morrison, 2017), VHs applications developed thus far serve more to fill gaps where a clinician is not available, than to aim at replacement of human providers. However, with anticipated advances in AI, the excitement and promise for developing these clinical VH systems needs to be balanced by a thoughtful and ethical concern for client safety and integrity.

## 9. Conclusions

Since the mid-1990s, VR-based testing, training, and treatment approaches have been developed that would be difficult, if not impossible, to deliver using traditional methods. At the same time, a large (but still maturing) scientific literature has evolved regarding the effects and outcomes from the use of VR targeting cognitive, psychological, motor, and functional impairments across a wide range of mental health conditions. What makes the clinical use of VR so distinctively important is that it represents more than a simple linear extension of existing computer technology for human use. By way of VRs capacity to immerse a user within an interactive computer-generated simulation, new possibilities exist that can go beyond the simple automation of previous clinical assessment and intervention approaches. Moreover, continuing advances in the underlying enabling technologies for creating and delivering VR applications have resulted in its recent widespread availability as a consumer product, sometimes at a very low cost. Thus, when one studies the scientific literature, examines the evolving state of the technology, and observes the growing enthusiasm for VR in the popular culture, the judgment for its future use in the area of trauma, while appealing, deserves more research attention. This is especially relevant when one considers the emotionally evocative and cognitively stimulating experiences that can now be produced in users of VR. Research is needed to understand the impact and effectiveness of this novel technology for specifying the extent to which VR is feasible and adds value for use with trauma populations.

This need for more research should not be surprising when one considers that the scientific study of human behaviour and interaction in the physical world has been the focus of psychology for about 125 years; it only makes sense that we may need a few more years to evolve the science for how humans behave and interact in the virtual world to better inform how we can safely and effectively use this technology with clinical populations.

In addition to VR’s potential for directly improving the efficacy of a clinical process, it may also serve as a tool for breaking down barriers to care. The main premise here is that the best evidence-based approach for assessing or treating a clinical health condition serves little value if clients do not seek it out and participate in it. There are many reasons why barriers to care limit client participation in clinical interventions that could ultimately provide benefit (Andrade et al., 2014; Clement, Schauman, Graham, Maggioni, & Evans-Lacko et al., 2015). To address this issue, we have constructed an intuitive model (Rizzo & Koenig, in press) for detailing and examining core barriers, referred to as the ‘7A’s’. The 7A’s refer the follow areas that are relevant targets for reducing such barriers: Awareness, Anticipated Benefit, Accessibility, Availability of well-trained providers, Acceptability of seeking treatment, Adherence, and Affordability. This model serves as a useful roadmap for discussing VRs potential impact on reducing barriers to psychological trauma care, while providing suggestions for future research.

Clinical VR may be strategically well-placed to break down some, but not all, of the barriers that keep people from receiving the benefits of evidence-based clinical care. To start, client *awareness* of the range of available treatment options may be limited. Perhaps some remedy for this exists in the media exposure that is currently at an all-time high for VR. In addition to the media excitement and interest in novel efforts to use VR for gaming and entertainment purposes, there has also been significant coverage of VR application in mental healthcare. This may be in part due to a desire in some quarters of popular culture to promote VR’s image as useful for pro-social purposes, beyond first person shooter games. Thus, a quick search of the internet will uncover a large volume of emotive media reports on VR’s application with clinical conditions, and this has been especially seen in the area of PTSD. Since the start of our trauma-focused VR research and development, the project has had over 400 popular media hits, many of which have occurred on highly visible mediums (https://www.youtube.com/playlist?list=PLMuMO5eoYy_A1CUIwMZ3Vy6E_GOMvg6Cd). Most recently, an actor using VRET for PTSD appeared as part of the narrative in the popular TV show, *House of Cards* (Strange, 2017). For better or worse, and in spite of the occasional scientific and factual errors in the popular press, there is no doubt that VR applications have received significant media visibility. Whether this enhances public awareness of treatment options that leads to actual help-seeking is still an open question in need of more research.

The media attention that is drawn to clinical applications of VR may also serve the value of promoting the *anticipated benefit* expected from its use, although this can be a double-edged sword. The balance between over-wrought claims of clinical success and actual data points in the popular media can sometimes err on the side of higher-than-warranted expectations. However, when research reports on clinical applications of VR do provide positive evidence, the popular media’s focus on covering those findings is fairly certain, thus reaching the eyes and ears of people who will hopefully seek help, either for themselves or a loved one. For example, our PTSD VRET exposure work has garnered significant popular media reporting that is typically followed by an uptick in client or family member queries as to where treatment can be accessed. The perception of the ‘sexiness’ of the use of ‘exotic’ VR technology in the popular culture may also build expectations of success that in the end may drive a stronger placebo effect in those who undergo VR-based services. And, at the very worst, once a client solicits help with a request for VRET and it is unavailable, the opportunity to offer them another form of evidence-based treatment may present itself, leading to treatment participation. However, this speculation would again require more research to confirm.

Making treatment more *accessible* is an important factor for promoting help-seeking in potential clients who live in remote locations or who encounter transportation or work scheduling challenges. This barrier has served to drive recent efforts at using PE teletherapy (Tuerk, Yoder, Ruggiero, Gros, & Acierno, 2010) or online self-help CBT programming (Olthuis et al., 2016). The similar use of VR as a tool for pushing trauma-relevant care outside of the clinic is still somewhat limited by cost and complexity issues, as well as by ethical concerns. The online or teletherapy delivery of trauma-focused VRET, although now technically feasible, will require further research to assure client safety and document effectiveness. However, applications that leverage a VH approach like SimCoach may be much closer on the horizon for providing remote support to trauma clients.

Another barrier to care concerns the *availability of well-trained providers* who are properly trained and certified in evidence-based trauma interventions (American Psychological Association, 2007). VRET has an added challenge in this area as it does require some specialized training in the operation of VR equipment. However, the impact of this is lessened by the fact that VRET follows the same procedures and mechanics as specified in the standard protocol for the delivery of imaginal PE (Foa, Hembree, & Rothbaum, 2007) and use of the equipment can be learned in a half-day training session. Such trainings are becoming more available from standalone workshops or CME offerings at relevant conferences. Alternatively, the use of VH patients to facilitate clinical expertise may offer a convenient and less labour intensive approach for providing experiential clinical training to reduce the impact of this barrier. While programmes that use virtual patients for clinical training are still in their infancy, this area is expected to grow in the near future as virtual human technologies are rapidly evolving.

The *acceptability* of seeking care can be improved by reducing the internal or external perceptions of stigma that a client may feel when seeking clinical help. This is an important issue, especially with trauma populations that have a more ‘macho’ self-perception where asking for help can be stigmatized as a sign of weakness (e.g. SMs and law enforcement officers) or for victims of sexual assault where perceptions of shame or self-doubt can inhibit care seeking. This is an area where clinical VR applications have some early research support. In a survey study to assess openness to seeking care in 325 active duty Army SMs (Wilson, Onorati, Mishkind, Reger, & Gahm, 2008), results indicated that 83% of the participants reported that they were neutral-to-very-willing to use some technology as part of a treatment; 71% were equally willing or more willing to use a treatment based on technology than to talk to a therapist in a traditional treatment setting. Moreover 20% of SMs, who stated they were not willing to seek traditional psychotherapy, rated their willingness to use a VR-based treatment as neutral to very willing. One possible interpretation of this finding is that a subgroup of this sample of SMs with a significant disinterest in traditional mental health treatment would be willing to pursue treatment with a VR-based approach. Thus, VR exposure therapy may offer an appealing treatment option for ‘digital generation’ SMs and Veterans who may be reluctant to seek out what they perceive as traditional talk therapies. Other research using VR exposure for PTSD and phobias with civilian groups have also shown high levels of treatment satisfaction (Baños et al., 2009; Beck, Palyo, Winer, Schwagler, & Ang, 2007) and, in some reports, college-age participants reported that it was easier to take the first step in confronting fears with VR compared imaginal exposure (Garcia-Palacios, Hoffman, Kwong See, Tsai, & Botella, 2001, 2007). From these results, one could speculate that younger groups who have grown up ‘digital’ may actually be more attracted to clinical VR treatments. Certainly, more research is needed to determine whether and how VR approaches reduce stigma and promote the acceptability of seeking care.

Promoting client *adherence* to a full course of trauma-focused treatment is a significant challenge with PTSD populations (Erbes, Curry, & Leskela, 2009). Although some small studies have suggested a higher positive interest in continuing treatment with VR (see Bryanton et al., 2006), most research examining treatment adherence in clinical VR applications have been underpowered. While the intrinsically engaging aspects of VR experiences are frequently referred to in the literature as motivating, we are not aware of any systematic evaluations of VR treatment characteristics and their impact on patient attrition for prolonged, repetitive treatment protocols. We speculate that as more story or narrative-based elements begin to appear in VR treatment applications (i.e. STRIVE), that client engagement in the dramatic elements of a personally relevant simulation could have a positive impact on adherence. However, understanding how to bridge the gap between the art and science for creating engaging VR treatment content and its effect on adherence is still an unexplored territory, worthy of further investigation.

*Affordability* has been an issue that has limited VR treatment access in the past. This is expected to be less of a limiting factor, now that higher fidelity, yet lower cost systems have come onto the market. As a point of comparison, it is now possible to purchase a high-fidelity VR HMD for US$600 (HTC Corporation, 2017) that has superior specifications compared to a system that five years ago would have cost US$20,000 (NVIS Inc., 2017). In addition, low-cost smartphone-based VR HMDs are likely to achieve parity with computer-tethered systems for some clinical VR applications, and this is predicted to dramatically reduce hardware costs and improve affordability. With large technology companies such as Facebook, Google, Apple, and Samsung invested in the VR market, we anticipate new and affordable hardware and software to be released more frequently over the next few years. As these companies continue their R&D work on innovative VR applications, we hope to see diversity and accessibility in this growing market, not unlike Google’s Play Store or Apple’s App Store, again with the result being more affordable prices for clinical end-users and eventually for clinician-supervised home use by patients.

In conclusion, we have detailed a range of applications that illustrate the current use of VR to address the behavioural healthcare needs of those suffering from the psychological effects of trauma. Since our work in this area was really instigated by the urgency to address the mental health needs of trauma-exposed SMs and Veterans from the OIF/OEF/OND combat theatres, it is only appropriate to put this work in a larger historical context. If one reviews the history of the impact of war on advances in clinical care, it could be suggested that clinical use of VR may be an idea whose time has come. For example, during World War I, the Army Alpha/Beta Classification Test emerged from the need for better cognitive ability assessment; that development later set the stage for the civilian intelligence testing movement over the next 40 years. Clinical psychology as a treatment-oriented profession was borne from the need to provide care to the many Veterans returning from World War II with ‘shell shock’ or ‘battle fatigue’ with the VA creating a clinical psychology intern programme in the late 1940s. At the same time, the creation of the National Institute of Mental Health (NIMH) came from an executive order from President Harry Truman as a vehicle for addressing the challenge of ‘Combat Neurosis’. More recently, the Vietnam War drove the recognition of PTSD as a definable and treatable clinical condition. In similar fashion, one of the clinical ‘game changing’ outcomes of the OIF/OEF/OND conflicts could derive from the military’s support for research and development to advance clinical systems that leverage new interactive and immersive technologies such as VR. This may drive wider uptake of clinical VR use in the civilian sector as the technology becomes more common in society’s digital landscape. Thus, as we have seen throughout history, innovations that emerge in military healthcare, driven by the urgency of war, typically have a lasting influence on civilian healthcare long after the last shot is fired.

However, such impact will only occur if positive efficacy and cost-benefit outcomes are generated from solid clinical research. As in all areas of new technology design and development, it is easy to get caught up in the excitement that surrounds the potential for innovative clinical opportunities, while ignoring the pragmatic challenges that exist for building and disseminating usable and evidence-based applications. Thus far, rational minds have prevailed among clinically-oriented VR developers and clinicians, most of whom have approached this area with an honest measure of enthusiastic vision, good science, and healthy scepticism. This has led to a growing interest in VR within the healthcare community, as clinical trials are incrementally demonstrating that VR can be implemented safely, at a reasonable cost, and that it has now begun to yield clinical outcomes that are at the least equivalent to, and sometimes more effective than, traditional approaches. Thus, any rush to adopt VR should not disregard principles of evidence-based and ethical clinical practice. In the end, technology is no more than a tool. The technology, in and of itself, does not ‘fix’ anyone. Rather, these systems are designed to either teach or extend the skills of a well-trained clinician and, in the case of SimCoach, to help a person to anonymously explore possible beneficial treatment options with a live human provider. Such clinical VR applications, while providing novel clinical options, will most likely produce therapeutic benefits when administered by a well-trained clinician with a professional appreciation of the complexity of these important behavioural healthcare challenges.

## Note

For those with further interest in the various Clinical VR application areas, here is a link to the complete collection of videos from the work of the USC ICT MedVR Lab: http://www.youtube.com/user/albertskiprizzo.
